# Regulator of calcineurin 1 (Rcan1) has a protective role in brain ischemia/reperfusion injury

**DOI:** 10.1186/1742-2094-9-48

**Published:** 2012-03-07

**Authors:** Mónica Sobrado, Belén G Ramirez, Fernando Neria, Ignacio Lizasoain, Maria Lourdes Arbones, Takashi Minami, Juan Miguel Redondo, María Ángeles Moro, Eva Cano

**Affiliations:** 1 Unidad de Neuroinflamación. Área de Biología Celular y del Desarrollo, Centro Nacional de Microbiología, Instituto de Salud Carlos III, 28220 Majadahonda, Madrid, Spain; 2 Unidad de Investigación Neurovascular. Departamento de Farmacología, Facultad de Medicina, Universidad Complutense de Madrid, 28040 Madrid, Spain; 3 Instituto de Biología Molecular de Barcelona (IBMB-CSIC) and Centro de Investigación, Biomédicas en Red de Enfermedades Raras (CIBERER), 08034 Barcelona, Spain; 4 Research Center for Advanced Science and Technology, the University of Tokyo, 153-8904 Tokyo, Japan; 5 Department of Vascular Biology and Inflammation, Centro Nacional de Investigaciones Cardiovasculares (CNIC), Melchor Fernández Almagro 3,, 28029 Madrid, Spain; 6 Unidad de Neuroinflamación. Área de Biología Celular y del Desarrollo, Centro Nacional de Microbiología, Instituto de Salud Carlos III Carretera Majadahonda-Pozuelo, Km.2,2,, Majadahonda 28220, Madrid, Spain

**Keywords:** Calcineurin, Calcium, Glia, Hypoxia, Inflammation, Rcan1, Stroke

## Abstract

**Background:**

An increase in intracellular calcium concentration [Ca^2+^]_i _is one of the first events to take place after brain ischemia. A key [Ca^2+^]_i_-regulated signaling molecule is the phosphatase calcineurin (CN), which plays important roles in the modulation of inflammatory cascades. Here, we have analyzed the role of endogenous regulator of CN 1 (Rcan1) in response to experimental ischemic stroke induced by middle cerebral artery occlusion.

**Methods:**

Animals were subjected to focal cerebral ischemia with reperfusion. To assess the role of Rcan1 after stroke, we measured infarct volume after 48 h of reperfusion in *Rcan1 *knockout (KO) and wild-type (WT) mice. *In vitro *studies were performed in astrocyte-enriched cortical primary cultures subjected to 3% oxygen (hypoxia) and glucose deprivation (HGD). Adenoviral vectors were used to analyze the effect of overexpression of Rcan1-4 protein. Protein expression was examined by immunohistochemistry and immunoblotting and expression of mRNA by quantitative real-time Reverse-Transcription Polymerase Chain Reaction (real time qRT-PCR).

**Results:**

Brain ischemia/reperfusion (I/R) injury *in vivo *increased mRNA and protein expression of the calcium-inducible Rcan1 isoform (Rcan1-4). I/R-inducible expression of Rcan1 protein occurred mainly in astroglial cells, and in an *in vitro *model of ischemia, HGD treatment of primary murine astrocyte cultures induced Rcan1-4 mRNA and protein expression. Exogenous Rcan1-4 overexpression inhibited production of the inflammatory marker cyclo-oxygenase 2. Mice lacking Rcan1 had higher expression of inflammation associated genes, resulting in larger infarct volumes.

**Conclusions:**

Our results support a protective role for Rcan1 during the inflammatory response to stroke, and underline the importance of the glial compartment in the inflammatory reaction that takes place after ischemia. Improved understanding of non-neuronal mechanisms in ischemic injury promises novel approaches to the treatment of acute ischemic stroke.

## Background

Stroke is a leading cause of human death and disability. However, despite the prevalence and consequences of brain ischemia the only effective treatment is to reinstate the blood supply, a course of action available in less than 3% of patients. Cerebral ischemia triggers a marked inflammatory reaction that involves local cellular activation in the brain and production of inflammatory mediators, including cytokines, chemokines, proteases, reactive oxygen species and vascular adhesion molecules (reviewed in [1]). Increased production of proinflammatory cytokines and chemokines has been detected in experimental models of brain ischemia, and in humans after stroke [2,3]. During focal ischemia, the cytokines interleukin 1β (IL-1β) and tumor necrosis factor α (TNFα) are generated very early and are secreted by cells within and around the injured territory [4]. Astrocytes are an efficient source of inflammatory mediators such as TNFα, granulocyte macrophage colony stimulating factor (GM-CSF), and others (reviewed in [5,6]). Expression of these factors can cause further activation of microglial, neuronal and endothelial cells, perpetuating immune/inflammatory signaling cycles if they are not halted by endogenous or exogenous anti-inflammatory agents.

One of the first events triggered by brain ischemia is an overload of intracellular calcium [Ca^2+^]_i _[7]. A key element of the cellular response to Ca^2+ ^signals is the phosphatase calcineurin (CN). The main mode of action characterized for CN is the regulation of the nuclear factor of activated T cells (NFAT) family of transcription factors (reviewed in [8,9]). Endogenous regulation of CN is mediated by members of the regulator of calcineurin (Rcan) family, previously named Down syndrome critical region (DSCR), modulatory calcineurin interacting protein (MCIP), calcipressin and Adapt78 in mammals [10,11]. The most studied Rcan member in mammals, and the only one regulated by Ca/CN, is Rcan1. There are two main Rcan1 protein isoforms, Rcan1-1 (252 amino acids) and Rcan1-4 (197 amino acids), resulting from differential promoter use and first exon choice [10]. Rcan1-4 is upregulated by increases in [Ca^2+^]_i _in several cell types, including brain cells, via a CN/NFAT-dependent pathway [12-16].

Rcan1 is highly expressed in brain [17], and elevated expression of Rcan1 transcript and protein in the brains of Down syndrome fetuses and Alzheimer patients has been described [18,19], as well as reduced Rcan1-1 expression in Huntington disease [19,20]. Rcan1 is also associated with oxidative stress [21,22], which together with its sensitivity to calcium suggests a possible implication in brain ischemia. Recent evidence indicates that Rcan1 is upregulated around the infarct area after experimental stroke [23]; however, the role of Rcan1 in the response to brain ischemia and calcium overload is unknown.

In the present work, we investigate the role of Rcan1 protein in response to brain ischemia. We report that Rcan1-4 protein and mRNA accumulate in brain cortex early after ischemia/reperfusion, mainly in glial fibrillary acidic protein (GFAP)-positive cells, and that Rcan1 deficiency worsens stroke outcome and increases expression of inflammation-associated genes. These findings suggest protective roles for Rcan1 during brain ischemia and neuropathologies with an inflammatory component.

## Materials and methods

### Models of transient focal cerebral ischemia

All animal procedures were performed in compliance with European Community law 86/609/ECC and were approved by the Ethics Committees of the Instituto de Salud Carlos III and the Universidad Complutense de Madrid.

For procedures in rat, adult male Fischer rats (275 to 300 g) were anesthetized with 1.5% isoflurane in a mixture of 70% nitrogen/30% oxygen. Rats in which the middle cerebral artery (MCA) and both common carotid arteries (CCA) were exposed but not occluded served as sham-operated controls. The femoral artery was cannulated for continuous monitoring of arterial pressure and blood sampling for analysis of pH, gases and glucose. Body and brain temperature was maintained at 36.5 ± 0.5°C throughout the procedure. Monitored physiological variables did not differ significantly between groups of animals before, during or after middle cerebral artery occlusion (MCAO) (data not shown). The surgical procedure was a variant of that previously described [24,25].

For procedures in mouse, 2-month-old adult male wild-type and *Rcan1 *knockout (KO) C57BL6 mice weighing 28 to 30 g were used. The KO strain has been described elsewhere [26], and was backcrossed into the C57BL6 background for more than 20 generations. Oligonucleotide primers used for genotyping analysis were 5'-GGTGGTCCACGTGTGTGAGA-3' and 5'-ACGTGAACAAAGGCTGGTCCT-3'. Mice were subjected to transient focal cerebral ischemia through a combination of both MCAO and ipsilateral common carotid artery occlusion (CCAO) for 90 minutes, followed by disocclusion of the two vessels and reperfusion for 48 h.

For protein extracts and RNA preparation, tissue samples were dissected from the ischemic brain region around the occluded MCA and from the corresponding non-infarcted region in the contralateral hemisphere and rapidly frozen. For immunohistochemistry, rats were deeply anesthetized (see above) 24 h after blood reperfusion and then perfused intracardially with 4% paraformaldehyde in phosphate buffer (PB, pH 7.4). The brains were removed immediately and post fixed overnight at 4°C in the same fixative. Brains were then cryoprotected by incubation in 30% sucrose in PB for 48 to 72 h at 4°C. Frozen coronal sections (50 μm) were cut with a freezing microtome (Leica, SM2000R) and processed for immunohistochemistry.

### Measurement of infarct volume

The infarct volume was estimated on Nissl-stained coronal sections using Cavalieri's principle [27]. Volume measurements were calculated using the 'contour' and 'Cavalieri estimator probe' of the Stereo Investigator software package. The spared tissue in the damaged hemisphere and the contralateral hemisphere were outlined. The software then randomly placed a quadratic grid of points over these areas. Each grid point thus represents an area. The program was used to estimate the area of the contours from the number of selected grid points. The associated volume was calculated by multiplying the area by the mean section thickness. The precision of the volume estimate for each brain was determined by computing the coefficient of error for the estimates. The ratio between the volume of the spared cortex in the damaged hemisphere (RN) and that in the whole tissue of the contralateral hemisphere (L) was calculated, and used to detect differences in the amount of cortex that was damaged by the infarct in each animal. When expressed as a percentage, this ratio indicates the percentage fraction of tissue spared from the ischemia; therefore the percent of neocortex that was infarcted (%I) is readily obtained by the formula %I = (1 - (RN/L)) ×100.

### Immunohistochemistry

Infarcted tissue in the neocortex was identified by staining slide-mounted coronal sections with Nissl (0.2% (w/v) cresyl violet). Adjacent sections were then processed for immunohistochemistry. Sections were permeabilized and blocked for 3 h at room temperature by incubation with 0.4% (v/v) Triton X-100 in Tris-buffered saline (TBS) containing 10% (v/v) normal serum from the species in which the secondary antibody was raised. Primary antibody was diluted in 0.2% (v/v) Triton X-100 in TBS containing 4% (v/v) of the same serum used for blocking. Goat polyclonal anti-GFAP antibody (Santa Cruz Biotechnology, Inc Santa Cruz, CA, USA, sc-6170) was diluted 1:500, and rabbit polyclonal Rcan1 antibody [26] was diluted 1:100. Sections were incubated with primary antibody overnight at 4°C. After several washes in TBS the sections were incubated for 2 h at room temperature with secondary antibodies: Cy3-conjugated anti-goat IgG or Alexa Fluor 488-conjugated anti-rabbit IgG, (Invitrogen, Grand Island, NY, USA, 1:200). After washing, sections were counterstained for 20 minutes at room temperature with TO-PRO 3 iodide (Molecular Probes) before mounting in Fluor-mount-G solution (Southern Biotech Birmingham, AL, USA). Parallel controls performed without primary antibodies showed very low levels of non-specific staining. Confocal images were acquired with a TCSSP5 confocal microscope (Leica Microsystems GmbH, Wetzlar, Germany).

### Cell culture and reagents

Cortical primary cultures enriched in astrocytes were prepared as described [28]. Cortical astrocyte cultures were plated at 4 × 10^5 ^cells per well in six-well plates or 1 × 10^6 ^cells per 10 cm^2 ^plate and grown to confluence, with medium changes every 3 days. After 17 to 20 days in culture, immunocytochemical analysis indicated that > 98% of the cells in the culture were GFAP positive.

The Ca^2+ ^ionophore A23187 was from Merck Biosciences Darmstadt, Germany. Phorbol 12myristate 13-acetate (PMA) was from Sigma-Aldrich St. Louis, MO, USA. Cyclosporin A (CsA) was from LC Laboratories Woburn, MA, USA..

### Hypoxia plus glucose deprivation (HGD) in vitro

Confluent primary astrocyte cultures were quiesced in Dulbecco's modified Eagle medium (DMEM) containing 0.5% fetal calf serum (FCS) overnight. Cultures were then subjected to hypoxia and deprived of glucose by incubation for 1 h in glucose-free DMEM (Life Technologies Corporation Carlsbad, CA, USA) plus 0.5% FCS in an anaerobic chamber (COY LaboratoryGrass Lake, MI, USA) in an atmosphere of 3% oxygen and 5% CO_2 _balanced with nitrogen. At the end of the required incubation period, cells were lysed and frozen inside the chamber.

### Cell lysis and immunoblot analysis

For whole-cell extracts, cells grown and stimulated in six-well plates or as indicated were washed twice with cold PBS and lysed for 30 minutes on ice in 100 μl hypertonic buffer with occasional mild agitation, as described [13]. Total tissue extracts were obtained by homogenizing the tissue in hypertonic buffer as before. Lysates were centrifuged for 10 minutes at 13,000 *g*, and the protein content of supernatants, containing cytosolic and most nuclear proteins, was quantified by Bio-Rad detergent-compatible protein reagent (Hercules, CA, USA). Total extracts were then boiled in 1× Laemmli buffer and resolved by 10% or 6% SDS-PAGE. Proteins were transferred to nitrocellulose membranes that were then immunoblotted as described [13]. The following antibodies were used: anti-NFATc3 polyclonal M75 (Santa Cruz Biotechnology, Inc Santa Cruz, CA, USA, sc-8321), monoclonal anti-α-tubulin (Sigma-Aldrich St. Louis, MO, USA, T8203), monoclonal Rcan1-4, raised and described by Minami *et al. *[15], and polyclonal Rcan1 antibody, raised and described by Porta *et al. *[26].

### RNA isolation, reverse transcription, and real-time PCR

Extraction of total RNA from rat or mouse cortical astrocyte cultures and analysis of differential gene expression by quantitative real-time Reverse-Transcription Polymerase Chain Reaction (real time qRT-PCR) were as described previously [13]. 18S rRNA and TATA-binding protein (TBP) transcripts were used as internal control genes and were amplified in the same tube to normalize for variation in input RNA. The amounts of target mRNA in samples was estimated by the 2^-ΔΔ*CT *^relative quantification method [29]. Ratios were calculated between the amounts of mRNA from stimulated and non-stimulated control cells.

### Adenoviral infection of astrocytes

The adenovirus bicistronically encoding green fluorescent protein (GFP) and human Rcan1-4 (Ad Rcan1-4) and the control adenovirus encoding GFP alone (Ad GFP) were as described [15]. Adenoviruses were generated and purified following standard protocols. Adenoviral infection was carried out on subconfluent primary astrocytes, and expression of the encoded protein was monitored by immunoblot.

### Calcineurin phosphatase activity assay

CN enzyme activity in brain tissue was analyzed with the Biomol Green Calcineurin Assay kit (Biomol, Plymouth, PA, USA) according to the manufacturer's instructions. Soluble protein extracts from three independent animals of each genetic background (Rcan1 wild-type (WT) and Rcan1 knockout (KO)) were prepared by homogenizing the tissue samples on ice in lysis buffer containing protein inhibitors. Soluble cytoplasmic proteins were prepared by ultracentrifugation (100,000 *g *for 45 minutes at 4°C). Excess sample phosphate was removed by passing samples through freshly prepared columns containing desalting resin (Bio-Rad, Hercules, CA, USA). To measure calcineurin phosphatase activity, protein extracts were quantified and equal protein amounts incubated for 30 minutes according to the assay kit instructions; liberated phosphate was measured colorimetrically at 620 nm. Calcineurin activity was calculated by comparison with a phosphate (PO_4_) standard curve.

### Statistical analysis

Results are expressed as the mean ± SEM or mean ± SD of the indicated number of experiments. Statistical analysis was by one-way analysis of variance (ANOVA) followed by individual comparisons of means (Student's t, Newman-Keuls or Bonferroni tests). Values were considered statistically significant at *P *< 0.05 (*) and *P *< 0.01 (**).

## Results

### Rcan1-4 protein is induced in murine models of brain ischemia/reperfusion injury

**Figure 1:**
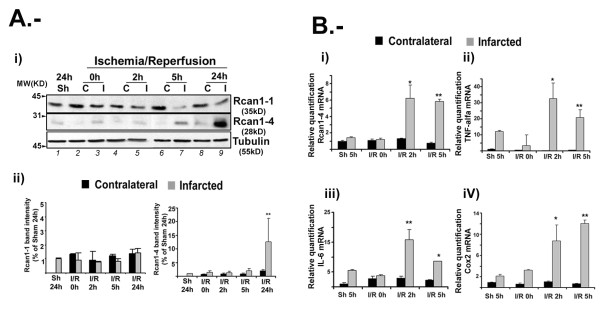
**Regulator of calcineurin (Rcan)1-4 protein and mRNA expression increase at sites of transient focal brain ischemia, paralleling increases in inflammatory cytokine mRNA levels**. **(A)**, panel (i) Representative immunoblot showing expression of endogenous Rcan1-1 and Rcan1-4 proteins in infarcted cortical tissue (I) and the corresponding region of the contralateral hemisphere (C) of rats subjected to middle cerebral artery occlusion (MCAO) for 1 h followed by reperfusion for the indicated times. Sh = sham operated animals. A total of 100 μg protein was analyzed per lane. Panel (ii), band density quantification from four independent experiments as in (A), normalized to α-tubulin. **(B) **Histograms showing relative quantification of mRNA expression by TaqMan real time qRT-PCR. Rat mRNAs for *Rcan1-4 *(i), *TNF α *(ii), *IL-6 *(iii) and *Cox-2 *(iv) were amplified from total RNA from infarcted and corresponding contralateral hemispheres of rats subjected to MCAO (1 h) followed by reperfusion (I/R) as indicated. Transcript amounts are normalized to 18S rRNA, and are expressed relative to contralateral samples from sham-operated animals after reperfusion for 5 h (Sh). Real time qPCR reactionwas conducted in triplicate for each condition, and data are the means ± SD of four experiments. **P < 0.01, *P < 0.05 (analysis of variance (ANOVA)) versus contralateral Sh samples.

The possible accumulation of Rcan1-4 protein in response to ischemia/reperfusion (I/R) brain injury was examined in a rat model. Ischemia was induced by MCAO for 1 h followed by reperfusion for varying periods up to 24 h. In this model, ischemia-induced necrosis is limited to the parietal and sensory-motor cortex, and large infarcts are reproducibly generated in the MCA territory. For each condition, the contralateral (C) hemisphere was analyzed in parallel to control for physiological events not specific to the infarcted (I) area. Tissue extracts from infarcted and contralateral areas were obtained, and the protein levels of the two main Rcan1 isoforms, Rcan1-1 and Rcan1-4, were measured by immunoblot. The level of Rcan1-4 in the infarcted area of the brain, where the occlusion took place, was increased relative to the contralateral area of sham-operated animals (Figure 1). Increased Rcan1-4 expression was noticeable after 5 h reperfusion and was very pronounced after 24 h (Figure 1A, panel i lane 9). Parallel analysis showed that in most cases the expression of the Rcan1-1 isoform varied only slightly throughout the I/R injury period (Figure 1A, panel i), although in some experiments a slight decrease in the levels of Rcan1-1 protein was seen in the infarcted area after the longer reperfusion periods (5 and 24 h). α-Tubulin protein levels generally remained constant, although a slight decline was observed in some experiments after 24 h reperfusion, possibly as a result of damage to the ischemic tissue (Figure 1B, panel ii).

### Transcript levels of Rcan1-4 and inflammatory cytokines are increased in the infarcted region after rat brain ischemia/reperfusion injury

*Rcan1-4 *mRNA expression in the same brain ischemia model was determined by real time qRT-PCR, together with the mRNA expression levels of the proinflammatory cytokines *IL*-*1β, TNFα *and interleukin 6 (*IL-6*) and the proinflammatory gene cyclo-oxygenase 2 (*Cox-2*). *Rcan1-4 *mRNA in the infarcted area was induced 2 h after I/R injury (Figure 1B), preceding the increase in Rcan1-4 protein; *Rcan1-4 *mRNA expression levels in the contralateral area were unchanged. Expression levels of the inflammatory markers in the infarcted area were also specifically increased after 2 h reperfusion and this increase was maintained at 5 h (Figure 1B, i to iv). A basal elevation in inflammatory marker expression in the infarcted area in the absence of I/R was probably due to meningeal membrane rupture during the experimental procedure. This increased inflammatory marker expression above the levels obtained in sham-operated animals agrees with published reports on other models of stroke (reviewed in [30]).

### Rcan1-4 is induced in GFAP positive astrocytes in vivo

**Figure 2:**
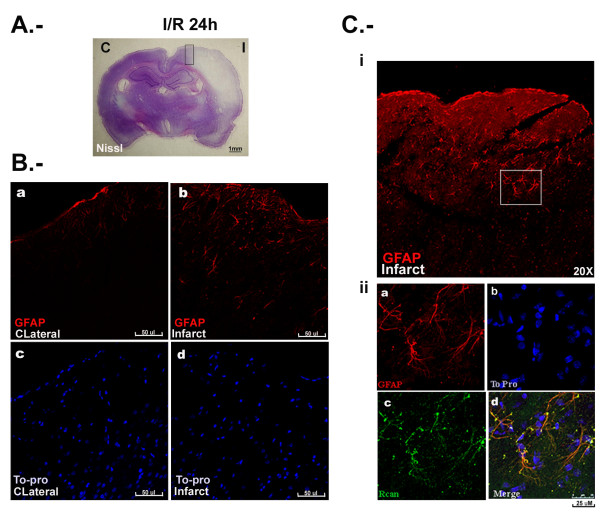
**Regulator of calcineurin 1 (Rcan1) expression colocalizes with glial fibrillary acidic protein (GFAP)-positive cells during transient focal brain ischemia**. Coronal brain sections of animals subjected to 1 h middle cerebral artery occlusion (MCAO) followed by 24 h blood reperfusion. **(A) **Nissl staining revealing a hypochromatic area of ischemic tissue in the infarcted (I) neocortex. **(B) **Immunohistochemistry showing expression of GFAP protein (red) in the contralateral hemisphere (a) and the infarcted region (b). Nuclei are revealed by staining adjacent sections with TO-PRO 3 (blue; c and d). **(C) **Immunohistochemistry showing the GFAP-positive area around the infarcted brain region. The boxed region is magnified in the lower panels: (a) GFAP immunostaining (red); (b) TO-PRO 3 nuclear staining (blue); (c) Rcan1 immunostaining (green); (d) overlay of (a-c). Yellow indicates GFAP-Rcan1 colocalization.

We next investigated the cellular location of the increased Rcan1 protein expression in response to brain I/R injury, using an anti-Rcan1 antibody that recognizes Rcan1-1 and Rcan1-4 isoforms [26]. After 24 h, Nissl staining of coronal sections detected a hypochromatic area in the infarcted neocortex, indicating neuronal injury (Figure 2A). The location of Rcan1 expression in relation to the injury site was analyzed by parallel immunohistochemical staining on adjacent sections. In addition, we stained for GFAP. In response to injury, astrocytes at the wound boundary migrate to repair the area. It is well established that astrocytes undergo a reactive process in response to different kinds of brain injury, such as ischemia and neurodegenerative diseases [6,31-33]. The reactive astrocyte phenotype is characterized by cellular hypertrophy, hyperplasia and increased expression of GFAP. At 24 h after early reperfusion, immunostaining with different anti-GFAP antibodies detected increased numbers of GFAP-positive cells in the neocortex surrounding the injured tissue (Figure 2B). Colocalization of Rcan1 in the majority of GFAP-positive cells around the infarct was confirmed by double immunostaining with goat anti-GFAP and rabbit polyclonal anti-Rcan1 antibodies (Figure 2C). These results agree broadly with a recent study describing Rcan1 protein accumulation in neuronal and astroglial cells, in which, as here, the antibody used for immunohistochemistry recognizes an Rcan1 C-terminal peptide common to both isoforms, [23].

### Cortical murine astrocytes express Rcan1-4 protein and mRNA in response to hypoxia plus glucose deprivation

**Figure 3:**
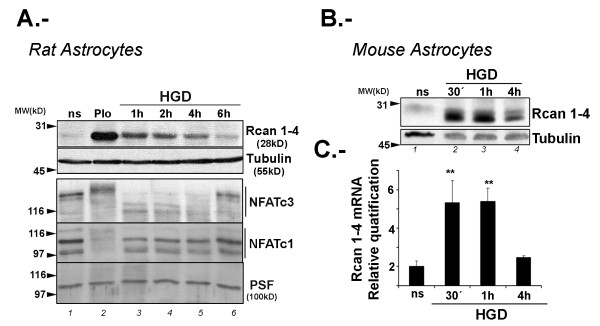
**Hypoxia (3%) and glucose deprivation induces regulator of calcineurin (Rcan)1-4 protein and mRNA expression in primary cortical murine astrocytes**. **(A, B) **Immunoblots showing endogenous Rcan1-4, nuclear factor of activated T cells (NFAT)c1 and NFATc3 protein expression, with α-tubulin and PSF (PTB-associated splicing factor) expression detected as loading controls. (A) Rat primary cortical astrocytes were non-stimulated (ns), treated with phorbol ester (phorbol 12-myristate 13-acetate) plus A23187 calcium ionophore (PIo) (4 h) as a positive control, or subjected to combined hypoxia (3% O2) and glucose deprivation (HGD) for 1 to 6 h. (B) Mouse primary astrocyte cultures were non-stimulated (ns) or subjected to HGD for 30 minutes to 4 h. **(C) ***Rcan 1-4 *mRNA was quantified by TaqMan real time qRT-PCR on total RNA from primary cortical mouse astrocytes stimulated as in (B). Transcript amounts are normalized to 18S rRNA and TATA-binding protein (TBP) endogenous controls, and are expressed relative to the level in non-stimulated control cells (ns). Data are the means ± SD of triplicate real time qRT-PCR determinations for each condition; n = 4. **P < 0.01 (analysis of variance (ANOVA)) versus ns.

To examine the effect of ischemia on astrocyte Rcan1-4 expression, highly pure cultures (> 98%) of primary murine astrocytes were subjected to periods of hypoxia (3% O_2_) combined with glucose deprivation (HGD). For comparison, cells were treated with phorbol ester (phorbol 12-myristate 13-acetate) plus calcium ionophore A23187 (PIo); Rcan1-4 expression in PIo-treated astrocytes is maximal after 4 h [28], although at this time NFATc3 protein appears as a slower migrating form (Figure 3A, lane 2). Compared with PIo, HGD provoked a more rapid and transient accumulation of Rcan1-4 protein, with levels increasing significantly within 1 h of HGD in rat and mouse astrocytes and beginning to diminish after 4 h (Figure 3A, B). A similar profile was observed for *Rcan1-4 *mRNA, with expression maximal after 60 minutes HGD, and diminishing to levels similar to those in non-stimulated cells after 4 h (Figure 3C). HGD treatment also induced dephosphorylation of NFATc3 protein, observed as a faster migrating band on immunoblot (Figure 3A).

**Figure 4:**
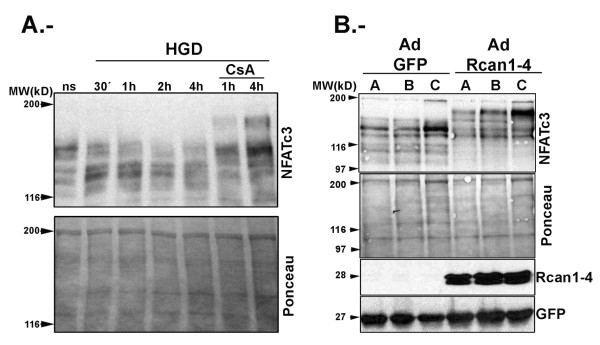
**Cyclosporin A (CsA) pretreatment and regulator of calcineurin (Rcan)1-4 overexpression cause the appearance of slower migrating nuclear factor of activated T cells (NFAT)c3 forms on SDS-PAGE**. **(A, B) **Total cell lysates were separated on 6% SDS-PAGE gels and immunoblotted with anti-NFATc3 antibody. Ponceau stainings of membranes are presented as loading controls. (A) Mouse primary astrocyte cultures were either left without pretreatment or were pretreated with CsA for 1 h as indicated. Cells then were left non-stimulated (ns) or were subjected to hypoxia and glucose deprivation (HGD) for 30 minutes to 4 h. (B) Three independent mouse astrocyte cultures **(A-C) **were infected with adenovirus bicistronically encoding green fluorescent protein (GFP) plus Rcan1-4 (Ad Rcan1-4) or encoding GFP alone (Ad GFP). Samples were run in parallel on 10% gels to analyze Rcan1-4 expression and to monitor GFP expression as a control of infection. Immunoblots show the effect of Ad Rcan1-4 infection on Rcan1-4 protein expression and NFATc3 electrophoretic mobility.

Western blot analysis of NFAT proteins is difficult to interpret, since most NFAT members exist as multiple isoforms. In the case of NFATc3, there are at least four mouse isoforms (according to the Ensemble database), with at least three of them in the range of 1060 to 1076 amino acids. These NFATc3 isoforms all contain the epitope used to raise the antibody used here; therefore to differentiate them we carried out immunoblot analysis on 6% SDS-PAGE gels, which were run until the 97 kDa marker approached the bottom of the gel in order to better resolve high molecular weight proteins (Figure 4A). This analysis shows that the antibody recognizes a number of bands that might correspond to the different mouse NFATc3 isoforms. To confirm that the bands we are studying correspond to NFATc3, we pretreated mouse astrocyte primary cultures for 1 h before HGD treatment with the calcineurin phosphatase inhibitor cyclosporin A (CsA). CsA prevented the appearance of the faster migrating bands after HGD treatment (Figure 4A). Furthermore, CsA changed the mobility pattern in non-stimulated cells toward slower migrating forms.

### Rcan1-4 protein expression modulates induced Cox-2 expression in primary astrocytes

**Figure 5:**
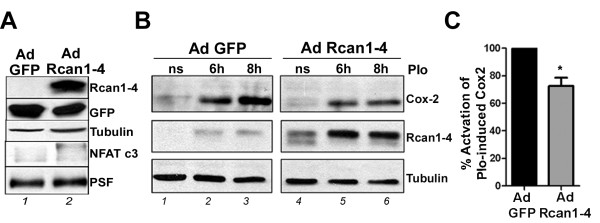
**Regulator of calcineurin (Rcan)1-4 protein downregulates expression of the proinflammatory gene *Cox-2 *in mouse primary astrocytes**. **(A) **Astrocytes were infected with adenovirus bicistronically encoding green fluorescent protein (GFP) plus Rcan1-4 (Ad Rcan1-4) or encoding GFP alone (Ad GFP). Immunoblots show the effect of Ad Rcan1-4 infection on Rcan1-4 protein expression and nuclear factor of activated T cells (NFAT)c3 electrophoretic mobility. α-Tubulin and PSF (PTB-associated splicing factor) were used as endogenous loading controls and GFP as a marker of equal infection. **(B) **Rcan1-4 overexpression inhibits astrocyte cyclo-oxygenase 2 (Cox-2) induction. At 48 h post infection, cells were quiesced, and then stimulated with phorbol ester (phorbol 12-myristate 13-acetate) plus A23187 calcium ionophore (PIo) as indicated. Blots show expression of Cox-2 and Rcan1-4 protein. Identical concentrations of adenoviral particles were used for each infection in all experiments. **(C) **Densitometry analysis of PIo-Cox2 induction. Data are the means ± SD of densitometry value of n = 4 experiments. The figure of 100% is the Cox2 expression achieved by PIo stimulation for 6 h in Ad GFP cells. PIo-induced Cox2 expression in Ad Rcan1-4 cells is 72 ± 11%. All values are corrected relative to the loading control α-tubulin.

To further analyze the potential role of Rcan1-4 protein in astroglial cell behavior, we infected three independent primary astrocyte cultures with an adenoviral vector bicistronically encoding Rcan1-4 plus green fluorescent protein (Ad Rcan1-4) or a control vector encoding GFP alone (Ad GFP) [15]. Total cell proteins were separated on 6% SDS-PAGE gels. Rcan1-4 overexpression in primary astrocytes caused the appearance of slower migrating forms of NFATc3 (Figure 4B), which match the NFATc3 forms observed in cells pretreated with CsA (Figure 4A). These results thus indicate that Rcan1-4 accumulation inhibits CN signaling by preventing NFAT dephosphorylation. Our previous work demonstrated that primary astrocytes respond to PIo treatment with a marked induction of Cox-2 protein expression [28]. Adenoviral overexpression of Rcan1-4 in primary astrocytes reduced the amount of Cox-2 induced by PIo by 30% (Figure 5B, C). Consistently, primary astrocytes deficient for Rcan1 expressed increased amounts of Cox-2 protein in response to this treatment (data not shown). These data are in line with the induced *Cox-2 *transcript expression in brain after I/R injury (Figures 1 and 5).

### Rcan1 gene deletion increases inflammatory gene expression and infarct volume after transient focal cerebral ischemia in mice

**Figure 6:**
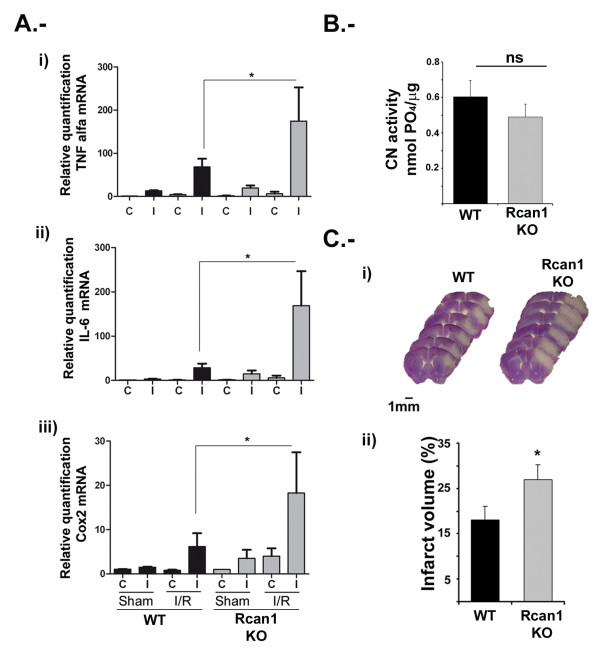
**Expression of ischemia/reperfusion-inducible inflammatory markers and infarct volume are increased in regulator of calcineurin (Rcan)1 knockout mice after transient focal cerebral ischemia in mice**. **(A) ***TNFα, IL-6 *and *Cox-2 *mRNAs were amplified by TaqMan real time qRT-PCR from total RNA obtained from infarcted (I) and corresponding contralateral hemispheres (C) of wild-type (WT) (black columns) and Rcan1 knockout (KO) (gray columns) mice subjected to 90 minutes middle cerebral artery occlusion (MCAO) followed by 5 h reperfusion. Transcript amounts are normalized to TATA-binding protein (TBP) as an endogenous control, and are expressed relative to the level in contralateral samples from sham-operated animals after reperfusion for 5 h (sham). Real time qPCR was conducted in triplicate for each condition, and data are the means ± SD of four experiments. **P < 0.01, *P < 0.05 (ANOVA) versus contralateral sham samples. **(B) **Calcineurin (CN) enzyme activity against phosphopeptide RII measured in brain cortices from *Rcan1 *WT and *Rcan1 *KO mice. ns = non-significant (Student's t test). **(C) **Mice were subjected to 90 minutes MCAO followed by 48 h blood reperfusion, and infarct volumes were estimated by Cavalieri's principle from Nissl-stained serial coronal sections. (i) Representative stacks of six Nissl-stained sections, revealing a larger hypochromatic area of ischemic tissue in the infarcted neocortex of Rcan1 KO animals. (ii) Quantification of cerebral infarct volume in WT and Rcan1 KO mice. The percentage of tissue volume infarcted in the right hemisphere = (1 - (R_N_/L) ×100), where RN is spared tissue in right (infarcted) hemispheres and L is the left (contralateral) hemisphere; cerebral tissue volume is expressed in mm^3^. Data are means ± SEM. n = 7 per genotype; *P < 0.05.

To investigate the precise role of Rcan1 expression after brain I/R injury we compared injured brain areas in WT and *Rcan1 *KO mice. For this, we first characterized the procedure for MCAO, previously described for rat, in the mouse. Transient focal cerebral ischemia (90 minutes) was produced in WT and KO mice in the C57/BL6 genetic background. In the brains of *Rcan1 *KO mice, I/R injury was accompanied by a more pronounced increase in the mRNA expression of inflammatory markers. After 5 h reperfusion, the increases in the expression of *TNFα, IL-6, IL-1β *and *Cox-2 *in WT mice were comparable to those encountered in the rat model (Figure 1 and 6). Infarcted cortical tissue extracts from Rcan1 WT and KO mice showed similar levels of CN activity toward the specific substrate RII phosphopeptide (Figure 6B), suggesting that Rcan1 deficiency does not significantly affect soluble CN phosphatase activity in brain cortices.

We next analyzed the functional consequence of *Rcan1 *deletion in this stroke model. The extent of I/R injury after 48 h reperfusion was determined from the infarct volume, measured from serial Nissl-stained sections. Our results show that infarct volumes were significantly larger in *Rcan1 *KO mice compared with WT (Figure 6). The MCAO procedure was not associated with animal death in either animal genotype.

## Discussion

In this study we report that Rcan1-4 protein and mRNA levels are increased after brain ischemia/reperfusion injury *in vivo*. Lack of Rcan1 is associated with larger infarct volume and higher expression of inflammation associated genes. *Rcan1 *expression after I/R injury occurs mainly in astroglial cells, correlating with the increased Rcan1-4 mRNA and protein expression observed in murine astrocytes subjected to hypoxia plus glucose deprivation. Consistent with the effect of Rcan1 deletion in the I/R *in vivo *model, overexpression of exogenous Rcan1-4 inhibits the production of the inflammatory marker Cox-2, while lack of Rcan1 augments this marker. These results support a protective role for Rcan1 during the inflammatory brain response during stroke.

Our immunoblot analyses indicate that the Rcan1 isoform induced after I/R is Rcan1-4 and not Rcan1-1 (Figure 1). Our data also show that the increased expression occurs mainly in GFAP-positive cells around the infarcted area. Using a different focal brain ischemia model, a recent report showed upregulation of Rcan1 protein in both the neural and glial compartments around the infarcted area [23]. This apparent discrepancy cannot be easily explained; however, in our experiments, in which staining was compared with the equivalent contralateral area, differential Rcan1 labeling was detected only in GFAP-positive cells. For our analysis of Rcan1 cellular localization in the rat MCAO I/R model we used a polyclonal antibody that recognizes both Rcan1-1 and Rcan1-4. While this antibody clearly distinguishes the two isoforms on immunoblots, from their different molecular masses, it cannot differentiate them by immunofluorescence staining. In our analyses, the Rcan1 antibody also stained NeuN-positive cells in the infarcted area, although this staining was no stronger than that observed in the contralateral area of sham-operated animals (data not shown). Thus increased Rcan1 reactivity was restricted to GFAP-positive cells, in agreement with the GFAP-positive staining seen by Cho *et al. *[23]. Moreover, the immunoblots indicate that the induced expression is due to Rcan1-4, with no significant change in Rcan1-1 (Figure 1). We therefore conclude that at the times examined the increased Rcan1 immunostaining in brain slices is due mostly to the expression of Rcan1-4 in glial cells surrounding the infarcted tissue. Confirmation of this must await the availability of Rcan1-1 and Rcan1-4 specific antibodies for immunohistochemistry analysis.

Treatment of astrocytes with PIo induced the appearance of a slower migrating band for NFATc3 protein (Figure 3A). Calcineurin activity has been classically estimated from the NFAT phosphorylation status [34,35]. We and others have shown that, in response to PIo, other NFAT proteins such as NFATc2 shift to a faster migrating band that has been considered the dephosphorylated form [13,36]. We also showed previously that NFATc1, c2, c3 and c4 are present in astrocyte cultures, with NFATc3 the most abundant member [28]. Pretreatment of these cells with CsA retards the gel mobility of all NFAT members analyzed. The unique response of NFATc3 to PIo induction has been reported previously by ourselves in astrocytes and by Urso *et al. *in Jurkat cells. These authors detected a slower migrating NFATc3 band at the same time (1 h) after exposure to PIo as we observe in astrocytes. This slower migrating form of NFATc3 might be a phosphorylated form generated by the action of PIo-induced kinases. Further experiments would be needed to demonstrate this hypothesis. To confirm that PIo induction in astrocytes efficiently translocates NFATc3 protein to the nucleus, we analyzed nuclear and cytosolic fractions (data not shown), detecting NFATc3 protein in the nuclear fraction of astrocytes treated with PIo for 1 h. When cells are pretreated with CsA, slower migrating NFATc3 forms were generated that run above 190 kDa; these NFATc3 forms accumulate in the cytosolic fraction and are not observed in the nuclear extract. Notably, non-stimulated astrocytes present NFATc3 proteins with intermediate mobilities on western blot analysis.

An anti-neuroinflammatory action of Rcan1 is consistent with inhibition of the CN signaling pathway. CN is a central inflammatory regulator in many cell types. In the brain, CN inhibitors such as CsA and FK506 have been shown to reduce tissue damage in response to stroke (reviewed in [37]). In line with our findings, Rcan1-4 expression has been shown to attenuate inflammatory and angiogenic responses [14,15,38], and a protective effect has also been proposed against oxidative stress [39]. Furthermore, targeted overexpression of constitutively active CN A in astrocytes has been reported to protect against brain inflammatory injury, as measured by negative regulation of inflammation hallmarks such as lipopolysaccharide-inducible Cox-2 and inducible NO synthase (iNOS) [40]. However, this study did not examine the expression of Rcan1-4, which is likely activated in response to the excess CN activity. Further studies to define the mechanism of endogenous modulation of CN activity will be important to determine how Rcan1 and CN influence the final outcome of the neuroinflammatory process.

Our current results with Rcan1 KO mice are compatible with Rcan1 downregulating the CN/NFAT signaling pathway. This notion is further supported by the appearance of slower migrating forms of endogenous NFATc3 in astrocytes overexpressing exogenous Rcan1-4 protein (Figures 4 and 5); these bands are very similar to the high molecular NFATc3 bands observed in CsA-treated cells (Figure 4). We suggest that these slower mobility forms might be hyperphosphorylated NFATc3 proteins. Furthermore, in cells overexpressing Rcan1-4 there is a partial inhibition of PIo-induced expression of the NFAT-dependent gene *Cox-2 *(Figure 5). The level of inhibition is similar to that obtained with CsA, as we previously described [28]. The potential of Rcan1 proteins to inhibit CN signaling either by an effect on CN phosphatase activity directly or by an action on the CN/NFAT signaling pathway via other means has been the focus of much controversy in the field. Rcan1 was originally identified as a negative regulator of CN activity [18,41-44]. However, other reports have shown that Rcan1 might not always repress CN activity. For example, heart tissue extracts form Rcan1 KO mice [45,46] and extracts from rcn1^-/- ^yeast (the Rcan1 homologue in yeast) [43], display reduced CN activity, leading these authors to conclude that Rcan1 proteins might facilitate CN activity. Biochemical analysis has shown that the specificity of signaling kinases and phosphatases toward their targets is usually mediated by docking interactions with substrates and regulatory proteins, and this is the case with Rcan1 and CN. Martinez-Martinez *et al. *recently showed that the inhibitory action of Rcan1 on calcineurin-NFAT signaling results not only from the inhibition of CN phosphatase activity, but also from competition between NFAT and Rcan1 for binding to the same docking site on calcineurin [47]. This competition for docking sites has been corroborated by another group [48]. Therefore the inhibition of the CN-dependent pathway might not be by direct inhibition of the activity of the phosphatase CN. We previously showed that CN is able to bind to endogenous Rcan1 proteins in astrocytes (see [28]), indicating that a similar mechanism might be operating in astrocytes. As we show in Figure 6B, Rcan1 loss of function does not affect CN enzymatic activity in brain cortices, indicating that in our system the inhibitory action of Rcan1 on CN-NFAT signaling is not due to direct inhibition of CN phosphatase activity.

The appearance of reactive gliosis in the periphery of the infarcted tissue is one of the most striking changes occurring in the brain after ischemia [49]. Furthermore, expression of constitutively active CN in astrocyte cultures has been shown to mimic the phenotype and gene transcription of activated astrocytes [50]. Although the exact role of this pathway in astrocyte activation is not completely understood, these findings suggest a scenario in which Ca^2+^-initiated CN activation is instrumental in the mounting of astrocyte inflammatory responses, with Rcan1-4 expression subsequently induced as an auto regulatory mechanism to prevent uncontrolled gliosis. It will therefore be of interest to study the effect of targeted glial-CN activation on the response to brain injury.

Although the precise mechanism of glial Rcan1-4 upregulation during brain ischemia is unclear, our results support an important contribution via hypoxia-induced CN-NFAT signaling. Little is known about the induction of CN-NFAT signaling by hypoxia, although transcriptional regulation has been reported in pulmonary arterial smooth muscle cells in response to chronic hypoxia [51] and in neuronal cells as a response to CN activation [52]. Rcan1-4 expression is a reliable marker of NFAT-dependent transcription, and our *in vitro *model of hypoxia plus glucose deprivation shows rapid (1 h) induction of Rcan1-4 expression and NFATc3 protein dephosphorylation. We have observed a clear inhibition of NFATc3 dephosphorylation by the CN inhibitor CsA (data not shown). We can therefore conclude that HGD activates the CN-NFAT signaling pathway in glial cells. However, other possible CN activators include proinflammatory cytokines such as IL-1β, TNFα, and IL-6, which are detected in the ischemic cortex 1 h after MCAO [53]. Indeed, IL-1β has been reported to activate the CN-NFAT pathway in highly enriched astrocytes [54]. In our hands, IL-1β only weakly induced Rcan1-4 expression in astrocytes, and we conclude that the initial Rcan1-4 expression in primary cultures is likely due to the calcium surge associated with the primary hypoxia. Consistent with an early, direct response to hypoxia, we see Rcan1-4 after 1 h in the *in vitro *astrocyte HGD model. However, the *in vivo *profile of Rcan1-4 expression in response to I/R injury is slower, with accumulation evident at 5 h and maximal expression detected at 24 h, the last time point examined. This profile probably reflects the complex multicellular environment of the intact brain, possibly including a contribution from the second wave of inflammatory cascades. The impact of other signaling pathways on Rcan1-4 expression during brain I/R injury is a possibility that should be pursued further.

## Conclusions

Inflammatory responses to ischemia can be mounted in response to one or various inflammatory stimuli, and the specific responses will vary for each cell type within the brain. Our data support a protective role for Rcan1 during the inflammatory response to stroke. Our results underline the need to take account of the impact of ischemia and its inflammatory consequences on the glial compartment, and to understand how glial cells respond to local ischemia in concert with the neurons they support. Improved understanding of non-neuronal mechanisms in ischemic injury promises novel approaches to the treatment of acute ischemic stroke.

## Competing interests

The authors declare that they have no competing interests.

## Authors' contributions

This study is based on an original idea from EC, who directed the work with MAM and IL. EC wrote the manuscript with the help of MAM, MS and IL. MS, MAM and IL provided expertise on ischemia/reperfusion animal models. MS carried out animal surgery and microscopy analyses and carried out the stereological and morphological studies. BR performed most of the biochemical studies with participation from EC and FN. TM and MLA provided animal models and reagents essential for this work. JMR made important conceptual contributions. All authors read and approved the final manuscript.
